# Annual Address before Connecticut Medical Society

**Published:** 1861-09

**Authors:** 


Addresses, Reports, etc.—Dr. Ashbel Woodward's annual address, as President of the Connecticut Medical Society, delivered at the convention of that body in May last, is an exceedingly practical essay on the comprehensive topic—*Life.* We have marked passages for reprint, which illustrate the speaker's axiomatic strength of style and treatment of his subject.

